# Supplementary material for: Structural and functional analysis of the GABARAP interaction motif (GIM)
Source: EMBO Rep. 2017 Jun 27;18(8):1382–96. doi: 10.15252/embr.201643587 (PMC5538626; doi:10.15252/embr.201643587)
Supplement: Supplementary file 1 — Appendix [file EMBR-18-1382-s001.pdf]

Table of Content

Appendix results

Thermodynamic analysis of interactions between PLEKHM1-LIR and autophagy modifiers. 2

Structural characterization of interactions between PLEKHM1-LIR and autophagy modifiers by NMR. 2

Understanding the contributing factors to PLEKHM1-LIR specificity towards GABARAPs. 4

Analysis of secondary structure elements for mutated in X_1 and X_2 positions PLEKHM1-LIR. 8

Appendix Tables

Appendix Table S1. Summary of crystal structure data collection and refinement statistics 11

Appendix Table S2. Angles between α2 and β2 in the mATG8s complexes with PLEKHM1 LIR. 12

Appendix Table S3. Distances (averaged across all monomers in an asymmetric unit (ASU)) between PLEKHM1-LIR residues (W635 and V638) and residues within mATG8-proteins. 13

Appendix Table S4. Thermodynamic parameters of interactions between PLEKHM1-LIR peptide and mutated LC3B and GABARAP proteins (K30R and R28K, respectively). 14

Appendix Figures

Appendix Figure S1. Structural analysis of the complexes between PLEKHM1-LIR peptide and mATG8 proteins. 15

Appendix references 17

Appendix Results

Thermodynamic analysis of interactions between PLEKHM1-LIR and autophagy modifiers.

The isothermal titration calorimetry experiments were aimed to provide affinities of interactions (K_D values) of all six human mATG8 proteins with synthetic PLEKHM1-LIR peptide, and to evaluate driving forces of the interactions (ΔH and -TΔS values) The K_D values (**Table 3**) indicate that PLEKHM1-LIR preferentially binds to GABARAP-subfamily proteins. K_D values for LC3s are in low µM range (3.45, 4.22 and 6.33 µM for LC3C, LC3A and LC3B, respectively) and decrease to sub-µM range for GABARAPs (0.55, 0.77 and 0.93 µM for GABARAP, GABARAP-L1 and GABARAP-L2, respectively). The binding enthalpy values (ΔH) of PLEKHM1-LIR interaction with all mATG8s are all favorable (negative values) and are significantly larger than those of NIX-LIR and OPTN-LIR interactions [1, 2], varying between -6 kcal/mol and -11 kcal/mol (**Fig. EV2A** and **Table 3**). However, the entropy contributions are distinct for each interaction. They vary from strongly favorable (positive values of ΔS) for LC3B to strongly unfavorable (negative ΔS) for GABARAP and LC3C (**Fig. EV2A** and **Table 3**). For the GABARAP and LC3C proteins, the ΔH/ΔS contributions are very close to the canonical LC3B interaction with p62-LIR [3], (**Fig. EV2A**). The variation of ΔS values reflects the importance of conformational flexibility in the formation of mATG8:PLEKHM1-LIR complexes, similar to the modulation of the observed flexibility for LC3B complexes with OPTN-LIR peptide in different phosphorylation states [4].

Structural characterization of interactions between PLEKHM1-LIR and autophagy modifiers by NMR.

We performed NMR titration experiments in order to find regions in the mATG8s proteins and PLEKHM1-LIR peptide that could drive the subfamily-specific interactions. First, we performed NMR experiments titrating ^{15}N-labelled LC3A, LC3B, GABARAP-L1 and GABARAP-L2 samples (as representative members of LC3- and GABARAP-subfamilies) with the PLEKHM1-LIR peptide. In agreement with our ITC data we observed “slow exchange” behavior of backbone HN resonances for the GABARAP subfamily proteins upon titrations. In this case, the two individual peak sets - “free” and “bound” - can be observed (**Fig. EV2B**). In contrast, behavior of backbone HN resonances for the LC3 subfamily proteins upon titrations with PLEKHM1-LIR peptide

represents an intermediate exchange mode where the peak maxima are gradually shifted from the "free" to "bound" position with the intensity decreasing upon addition of the PLEKHM1-LIR peptide (**Fig. EV2B**). Overall, this indicates that PLEKHM1-LIR binds GABARAP-L1 and -L2 proteins more tightly than LC3A and LC3B proteins.

We mapped the chemical shift perturbations (CSP) on the sequences (**Fig. EV2C**) and structures (**Fig. EV2D**) of all four proteins used in this experiment, revealing a high degree of similarity in the CSP patterns. Most affected are the backbone HN resonances of residues forming the hydrophobic pockets 1 and 2 (HP1 and HP2, highlighted on **Fig. EV2D**) and β-strand 2 which participates in formation of the intermolecular β-sheet between mATG8 proteins and the LIR sequences [3-7]. However, binding of the PLEKHM1-LIR peptide induces more concise CSPs around and inside of the GABARAP-L1 and -L2 hydrophobic pockets, whereas CSPs for LC3A and LC3B are additionally seen on the external side of helix α2 near HP1. In addition, PLEKHM1-LIR binding to GABARAP-L1 and –L2 affects residues within helix α3 near HP2 compared to the LIR binding to LC3A and LC3B (**Fig. EV2D**). We also observed some differences in CSP patterns for individual proteins, indicating that there might be subfamily-specific structural and dynamic background for preferential PLEKHM1-LIR binding. In particular, residues in loop 3 connecting strands β1 and β2 show a higher degree of structurization for the GABARAP-subfamily upon binding the PLEKHM1-LIR peptide. In contrast residues within loop 6 connecting strand β3 and helix α4, show a higher degree of structurization in LC3 subfamily complexes.

We complemented these NMR titration experiments by titration of ^{15}N labeled PLEKHM1-LIR peptide with non-labelled LC3B WT and GABARAP WT proteins to find which residues within PLEKHM1-LIR peptide are most affected by interactions with LC3s and GABARAPs (**Fig. EV2E**). NMR titrations revealed that with both LC3 and GABARAP, the most pronounced CSP of backbone HN proton resonances for PLEKHM1-LIR peptide were detected for the residues within core LIR motif - V636, N637 and V638 (**Fig. EV2E**, upper plots) and Hδ2 side-chain resonances N637 (**Fig. EV2E**, lower plots). Interestingly, we observed only slight shifts of the HN backbone resonances for acidic residues in positions -3, -2 and -1 (E632, D633, D634) prior to the core LIR motif and a small shift of W635 backbone HN resonance (**Fig. EV2E**). In contrast, W635 side-chain

Hε1 resonance undergoes strong perturbation according to the "slow"-exchange mode. This resonance serves as a reporter for the state of the tryptophan aromatic ring, which is deeply inserted into HP1 upon mATG8 binding, and is visible at each titration step in its "free" and "bound" form (**Fig. EV2B**, right plots). In agreement with our crystal structures, we observed large CSPs for δ2 H_2N- resonances of X_2-occupied N637 (**Fig. EV2E**, upper plots), indicating that this side chain could play an important role in coordination of PLEKHM1-LIR on surface of mAtg8 proteins.

Understanding the contributing factors to PLEKHM1-LIR specificity towards GABARAPs.

We determined the crystal structures of the LC3A, LC3C, GABARAP and GABARAP-L1 proteins in complex with the PLEKHM1-LIR to probe the molecular reasons why this LIR peptide binds with higher affinity to the GABARAP-subfamily, as compared to the LC3-subfamily of proteins (**Figure 2A**). The overall complex structures are presented in **Appendix Figure S1**, together with omit maps for each complex structure, verifying that the peptides are bound. Data collection and structural refinement statistics for all these complexes can be found in **Appendix Table S1**. The monomers within the asymmetric unit of each PLEKHM1-LIR complex overlaid well (PLEKHM1-LIR:LC3A, r.m.s.d. = 0.174 Å; PLEKHM1-LIR:LC3B, r.m.s.d. = 0.217 Å; PLEKHM1-LIR:GABARAP, r.m.s.d. = 0.167 Å; PLEKHM1-LIR:GABARAPL1, r.m.s.d. = 0.326 Å; PLEKHM1-LIR:LC3C co-crystal complex, r.m.s.d. = 0.426 Å) (**Fig. EV3A**). Thus, the monomer A within the asymmetric unit of each structure was used in the analysis.

Here we provide a detailed comparison across the LIR bound and unbound mATG8 structures, starting with global differences and then focusing on the LIR binding surface.

1) Analysis of the global changes in protein conformation upon LIR binding across the GABARAP- and LC3-subfamilies suggest only subtle differences that do not, by themselves, explain the altered binding affinity.

i) *Altered angles between helix α2 and β-strand β2 between the GABARAP and LC3 families*. The orientation of the α-helix 2 shows a clear separation between the GABARAP and LC3 subfamily members (**Fig.s EV3C and EV3D**). The angle between α2 and β2 for GABARAP is -34.2° while the same angle is -43.4° for LC3B (**Appendix Table S2**). We note that a smaller angle

between both secondary structure elements correlates with a tighter binding between the LIR and mATG8 proteins: for the GABARAP proteins (K_D ranges from 0.55-0.77 μM) the angle is ~35°, whereas the for the LC3-subfamily of proteins (K_D ranges from 3.5-6.3 μM) the angle is ~41°. However, this may be influenced by considerable crystal packing contacts through the α2 helix that are present in the LC3 structures, but not the GABARAP structures.

ii) Altered conformation of loop 3. The conformation of loop 3 (β-1-β2; **Fig. EV3E**) is different across the two families, which is likely caused by the insertion of an extra residue within the LC3-subfamily of proteins (loop comprises 14 residues in the LC3-subfamily, but 13 residues in the GABARAP-subfamily). The loop is largely distal to the LIR binding surface, although the C-terminal end of this provide residues K49 and K51 (LC3A numbering), which are important for LIR binding. However, an alignment of the structures demonstrates that the conformation at the C-terminal end of the loop is the same across the mATG8 structures.

iii) Altered conformation and dynamics of loop 6. For loop 6, which is on the face opposite from the LIR binding site, the conformation is variable across the GABARAP and LC3 subfamilies (β3-α4; **Fig. EV3F**). As in loop 3, structural overlays of monomer A in each crystal structure demonstrate that the length of loop 3 is one residue shorter for the GABARAP-subfamily (11 residues) versus the LC3-subfamily of proteins (12 residues). However, despite Loop 6 being located distal to the HP1 and HP2 pockets, it is significantly altered upon LIR binding to the LC3 proteins, but not the GABARAP proteins (**Fig. EV4G**). According to our NMR experiments, in LC3-proteins Loop 6 switches between unstructured (LIR-unbound; low signal intensities) and structured (LIR-bound; increased backbone HN resonance intensity), resulting in significant CSP for the HN resonances within the loop. However, for GABARAP-proteins, the corresponding CSP are small, indicating this loop remains mostly structured in both unbound and LIR-bound state, highlighting differences between these related, but different families of mATG8-proteins.

2) Detailed analysis of difference in the LIR binding surface.

i) *Analysis of the Θ position.* This LIR-position is occupied by PLEKHM1 W635, the largest and most common LIR aromatic residue in this position. In the LIR-bound state of the GAPARAP

proteins, the W635 side chain fits tightly into the hydrophobic pocket 1 (HP1), making a number of hydrogen bonds and hydrophobic interactions (**Figure 3A**), whereas the distances corresponding for these interactions are noticeably longer for the LC3 proteins (see **Appendix Table S3** for detailed analysis of the interaction distances). Oε1 of residue E17 in GAPARAP and GAPARAPL1 is 3.5 and 4.3 Å (mean across all monomers in the ASU) away from the aromatic Nε1 of W635, providing a possibility to form the intermolecular hydrogen bond. In comparison, the corresponding atoms are further apart in the LC3-family complexes, especially LC3B (Oδ1 of residue D19 to Nε1 of W635 - 6.2 Å) and LC3C (Oε1 of residue E25 to Nε1 of W635 - 4.5 Å). In LC3A, this distance is comparable with that for the GABARAP-subfamily (Oε1 of residue E25 to Nε1 of W635 - 3.5 Å). Additionally, aromatic carbons of W635 are significantly closer to the carbons of GABARAP non-polar residues, forming the HP1 (**Figure 3A** and **Appendix Table S3**). For example, distances between CZ2 of PLEKHM1 W635 to CZ of GABARAP and GABARAP-L1 F104 are 3.5 and 3.7 Å, respectively; while for to the corresponding CZ of LC3A F108 - 4.0, CZ of LC3B F108 - 4.2 and CZ of LC3C F114 - 4.5 Å, respectively. Overall, the conformation adopted by W635 of PLEKHM1 is considered more optimal for the interaction with GABARAP-subfamily, compared to the LC3-subfamily, proteins.

ii) Analysis of the X_1 position. Structural analysis of PLEKHM1-LIR V636, occupying X_1 position, did not revealed significant subfamily-specific differences in the distances of this residue to the closes residues within mATG8 proteins. For both LC3- and GABARAP-subfamily proteins, the hydrophobic surface that accommodates V636 is lined with 1) phenylalanine (F52/F52/F58 for LC3A/B/C) or tyrosine (invariant Y49) for the GABARAPs; 2) the aliphatic moiety of lysine side chain (K51/K51/K57 for LC3A/B/C; K48 for GABARAPs), where the amine group is exposed into solution, and 3) the invariant for all mATG8 arginine side chains (R70/R70/R76 for LC3A/B/C, and R67 for GABARAPs). For LC3-subfamily proteins we found that R70/76 forms a salt bridge with invariant D48/D48/54 in LC3A/B/C side chain, which holds the arginine in a fixed conformation **(Fig. EV4A**, left plot). In contrast, such a salt bridge is absent in the GABARAP-family proteins, the D45 side chain is disordered (not visible in the electron density maps), resulting in the “disordering”

of the arginine side chain leading to a less favorable packing of V636 on surface of GABARAP-family proteins (**Fig. EV4A**, right plot).

iii) Analysis of the X_2 position. Inspection of our mATG8:PLEKHM-LIR structures for residues interacting with N637 (position X_2) shows that the Oγ of N637 is in an ideal position for hydrogen bonding to the conserved arginine (R28) in the GABARAP-subfamily proteins. For the LC3-subfamily proteins, however, the N637 side chain carboxyl is not in an ideal position for intermolecular hydrogen bonding (**Figure 3C**). Additionally, residues R28 are in an ideal position to make a T-type cation-π interaction [8] with a GABARAP-conserved tyrosine (Y25), which may enhance the intermolecular hydrogen bond by placing the side chain of arginine in an ideal position (**Fig. EV4B**). In the LC3-subfamily proteins, this is a histidine in LC3A and LC3B (H27), or phenylalanine in LC3C (F33), and the lysine residues do not form a cation-π interaction with these side chains in all the LC3:PLEKHM1-LIR complexes. To test whether the difference in these hydrogen bonds correlate with the difference in specificity of GABARAP- and LC3-families towards PLEKHM1-LIR, we substituted R28 in GABARAP to lysine and K30 in LC3B to arginine and performed comparative ITC and NMR titration experiments with mutated proteins and PLEKHM1-LIR peptide.

We titrated LC3B K30R and GABARAP R28K mutants with PLEKHM1-LIR peptide and compared the ITC data with that for the LC3B WT and GABARAP WT proteins. Notably, there is no significant difference between K_D values of PLEKHM1-LIR interactions with wild type proteins and K30R/R28K mutants (**Fig. EV4C** and **Appendix Table S4**). Nonetheless, we did observe a small increase in favorable binding enthalpy for LC3B K30R mutant (0.3 kcal/mol); and smaller favorable enthalpy for GABARAP R28K (0.05 kcal/mol) that could be interpreted as a stabilization of mATG8-proteins complexes with PLEKHM1-LIR over the more favorable arginine at position 28 in GABARAPs (**Appendix Table S4**). However, these small changes are within experimental error and too small to serve as a determinant of specificity.

NMR titrations of ^{15}N labeled PLEKHM1-LIR peptide with non-labelled LC3B K30R and GABARAP R28K confirmed ITC data. Indeed, the LC3B K30R mutant induced stronger CSP for side-chains resonances of N637 in NMR titration experiments; and GABARAP R28K induced a smaller CSP

for side-chains resonances of N637 (**Fig. EV4D**, lower plots, blue contours). Also backbone HN resonances of N637, and adjacent V636 and V638 residues undergoes larger CSP upon binding LC3B K30R mutant (in comparison to LC3B WT), while binding of GABARAP R28K mutant induces smaller CSP (in comparison with GABARAP WT). Other backbone HN resonances remain insensitive to the mutations (**Fig. EV4D**). Therefore, based on our ITC and NMR results, we propose that N637 of PLEKHM1-LIR is important for the stabilization of PLEKHM1-LIR interaction with GABARAP, rather than being a defining factor in determining LC3 or GABRAP specificity.

iv) Analysis of the Γ position. Compared to the HP1 pocket, the HP2 pocket is shallow (**Fig. EV5B**). The key interactions for this pocket are 1) a main-chain hydrogen bond between the nitrogen of PlekHM1 V638 and oxygen of an invariant LC3/GABARAP leucine, which mediates formation of the intermolecular β-sheet, common for all canonical LIR:ATG8-proteins interaction (reviewed in [9]), and 2) hydrophobic interactions between the sidechain and the hydrophobic residues that line the HP2 pocket (**Figure 3D** and **Appendix Table S3**). Judging by the bonding distances (**Appendix Table S3**), V638 of the PLEKHM1-LIR fits more tightly within the HP2 pocket of the GABARAP-subfamily proteins, as compared to the LC3-subfamily proteins. The sidechain of V51 in the GABARAP proteins is in closer proximity to the PLEKHM1 V638 (GABARAP = 3.8 Å and GABARAP-L1 = 4.3 Å), while side chains of residues in equivalent positions of LC3-subfamily proteins are further away (LC3A/B/C V54/V54/V60 - 4.2/4.6/4.6 Å). Similarly, the L55 sidechain in the GABARAP proteins is closer to the PLEKHM1 V638 (GABARAP = 4.6 Å and GABARAP-L1 = 4.3 Å), as compared to the LC3 proteins (LC3A V58 = 5.5 Å, LC3B V58 = 5.4 Å, LC3C L64 = 4.3 Å). As in the HP1 pocket, the bonding distances (particularly the mean distances of equivalent positions) of the hydrophobic interactions in the HP2 pocket are shorter in the GABARAP proteins.

Analysis of secondary structure elements for mutated in X_1 and X_2 positions PLEKHM1-LIR.
In order to evaluate the role of small (G;A,P,S) and positively-charged (K,R,H) residues at PLEKHM1-LIR positions X_1 and X_2 with respect to their ability to adopt a β-stranded conformation and thus participate in formation of the intermolecular β-sheet, we predicted secondary structure elements within a relatively short stretch of PLEKHM1 residues (26-mers, PLEKHM1 residues 625-

650, wild type and mutated sequences) by JPRED4-server ([10], http://www.compbio.dundee.ac.uk/jpred4). In all but one cases, mutations in positions X_1 and X_2 do not affect significantly probability of the PLEKHM1 26 residues long sequence to form β-strand near W635. The only X_1 V636P mutant is predicted as unable to form the β-strand. Generally, all residues at position X_1 participates in formation of intermolecular β-sheet with both backbone amino- (HN) and carboxy- (CO) groups, forming two intermolecular hydrogen bonds towards β-strand β2 in the mATG8 molecule (to the backbone of F52/F58/Y49 across both the LC3 and GABARAP families, Figure 3B). Therefore, presence of proline residue in this position is definitely unfavorable for the canonical LIR:mATG8 interaction and therefore proline in position X_1 is not used in in the natural LIR sequences. Interestingly, there is a natural LIR, containing Pro residue in X_1 position - β-catenin LIR (504-W**P**LI-507) described in [11]. This Pro residue is evolutionary conserved as well as the whole β-catenin sequence around LIR (see below). Secondary structure prediction indicates the β-catenin residues within and near this potential LIR motif form an α-helix (in fact, it is a part of α-helical armadillo repeat domain), thus, this LIR probably interacts with mATG8 via not canonical mechanism involving bigger than short LIR-motif protein-protein contacts. However, for other residues at this position there are no limitations with respect to intermolecular hydrogen bonding; also for position X_2 all residues are equally capable to adopt β-conformation. Therefore, we can conclude that the effect of these substitutions on the mATG8 proteins binding (**Fig. EV5C** and **EV5D**) is mediated by specific sidechain structure, orientation and mobility, and not by ability of mutated PLEKHM1-LIR adopts β-stranded conformation.

Results of secondary structure prediction for mutated PLEKM1-LIR:

1. PLEKHM1-LIR WT

```
                         : ---630-------640-------650 :
OrigSeq                  : QKVRPQQEDEWVNVQYPDQPEEPPEA :
Jnet                     : ----------EEEE------------ :
Jnet Rel                 : 89887777731762056776777889 :
```

2. PLEKHM1-LIR X1 mutants

2.1 V636G

```
                         : ---630-------640-------650 :
OrigSeq                  : QKVRPQQEDEWGNVQYPDQPEEPPEA :
Jnet                     : -----------EE------------- :
Jnet Rel                 : 89887777630213677766777889 :
```

2.2. V636P

```
                         : ---630-------640-------650 :
OrigSeq                  : QKVRPQQEDEWPNVQYPDQPEEPPEA :
Jnet                     : -------------------------- :
```

```
Jnet Rel                : 89887777653256677667777889 :
```

2.3. V636K

```
                        : ---630-------640-------650 :
OrigSeq                 : QKVRPQQEDEWKNVQYPDQPEEPPEA :
Jnet                    : ----------EEE------------- :
Jnet Rel                : 89887777730312156776777889 :
```

2.4. V636R

```
                        : ---630-------640-------650 :
OrigSeq                 : QKVRPQQEDEWRNVQYPDQPEEPPEA :
Jnet                    : -----------E-------------- :
Jnet Rel                : 89887777630203677766777889 :
```

2.5. V636H

```
                        : ---630-------640-------650 :
OrigSeq                 : QKVRPQQEDEWHNVQYPDQPEEPPEA :
Jnet                    : -----------EE------------- :
Jnet Rel                : 89887777730200356666777889 :
```

3. PLEKHM1-LIR X2 mutants

3.1. N637G

```
                        : ---630-------640-------650 :
OrigSeq                 : QKVRPQQEDEWVGVQYPDQPEEPPEA :
Jnet                    : ----------EEE------------- :
Jnet Rel                : 89887777630410156666777889 :
```

3.2. N637P

```
                        : ---630-------640-------650 :
OrigSeq                 : QKVRPQQEDEWVPVQYPDQPEEPPEA :
Jnet                    : ----------EEEE------------ :
Jnet Rel                : 89887777731651156666777889 :
```

3.3. N637K

```
                        : ---630-------640-------650 :
OrigSeq                 : QKVRPQQEDEWVKVQYPDQPEEPPEA :
Jnet                    : ----------EEEE------------ :
Jnet Rel                : 89887777731762056776777889 :
```

3.4. N637R

```
                        : ---630-------640-------650 :
OrigSeq                 : QKVRPQQEDEWVRVQYPDQPEEPPEA :
Jnet                    : ----------EEEE------------ :
Jnet Rel                : 89887777731762056776777889 :
```

3.5. N637H

```
                        : ---630-------640-------650 :
OrigSeq                 : QKVRPQQEDEWVHVQYPDQPEEPPEA :
Jnet                    : ----------EEEE------------ :
Jnet Rel                : 89887777731762056776777889 :
```

Conservation of β-Catenin LIR motif residues

```
P35222  CTNB1_HUMAN   481 AQNAVRLHYGLPVVVKLLHPPSHWPLIKATVGLIRNLALCPANHAPLREQGAIPRLVQLL 540
Q0VCX4  CTNB1_BOVIN   481 AQNAVRLHYGLPVVVKLLHPPSHWPLIKATVGLIRNLALCPANHAPLREQGAIPRLVQLL 540
Q02248  CTNB1_MOUSE   481 AQNAVRLHYGLPVVVKLLHPPSHWPLIKATVGLIRNLALCPANHAPLREQGAIPRLVQLL 540
B6V8E6  CTNB1_CANLF   481 AQNAVRLHYGLPVVVKLLHPPSHWPLIKATVGLIRNLALCPANHAPLREQGAIPRLVQLL 540
P26233  CTNB_XENLA    481 AQNAVRLHYGLPVVVKLLHPPSHWPLIKATVGLIRNLALCPANHAPLREQGAIPRLVQLL 540
Q90424  Q90424_DANRE  480 AQNAVRLHYGLPVVVKLLHPPSHWPLIKATVGLIRNLALCPANHAPLREQGAIPRLVQLL 539
                          ************************************************************
```

Results of secondary structure prediction for β-Catenin LIR motif:

```
                 : ----500-------510--------- :
OrigSeq          : VVKLLHPPSHWPLIKATVGLIRNLAL :
Jnet             : -----------HHHHHHHHHHHH--- :
Jnet Rel         : 86578888752789999999853399 :
```

Appendix Table S1: Summary of crystal structure data collection and refinement statistics.

	**PLEKHM1-LIR: LC3A (PDB: 5DPR)**	**LC3C: PLEKHM1-LIR* (PDB: 5DPW)**	**PLEKHM1-LIR: GABARAP (PDB: 5DPS)**	**PLEKHM1-LIR: GABARAP-L1 (PDB: 5DPT)**
Data-collection statistics				
Space group	$P2_1$	$P1$	$P3_1$	$P3_221$
Cell parameters				
a, b, c (Å)	62.7, 63.1, 84.9	54.1, 73.2, 89.1	73.8, 73.8, 74.5	84.8, 84.8, 105.4
α, β, γ (°)	90, 100.9, 90	89.96, 89.99, 77.3	90, 90, 120	90, 90, 120
Wavelength (Å)	0.9537	0.9537	0.9537	0.9537
Resolution (Å)	45.57-2.50 (2.60-2.50)	38.61-2.19 (2.24-2.19)	37.23-2.00 (2.05-2.00)	42.8-2.90 (3.03-2.90)
R_{merge}	0.113 (0.614)	0.074 (0.875)	0.196 (3.387)	0.148 (2.352)
$R_{p.i.m.}$	0.068 (0.366)	0.048 (0.573)	0.060 (1.066)	0.048 (0.735)
$R_{meas.}$	0.132(0.716)	0.089 (1.063)	0.205 (3.652)	0.156 (2.466)
I/σI	7.1 (2.1)	13.4 (1.9)	10.3 (1.5)	10.6 (1.4)
Completeness	100 (100)	98.6 (97.8)	99.2 (98.6)	99.9 (99.5)
CC(1/2)	0.928 (0.754)	0.999 (0.824)	0.998 (0.651)	0.991 (0.581)
Redundancy	3.8 (3.8)	6.6 (6.6)	11.6 (11.6)	10.9 (11.0)
Total reflections (Unique)	22,780 (2,562)	446,563 (68,176)	353,770 (26,146)	110,277 (17,825)
Refinement statistics				
R_{work}/R_{free} (%)	21.8/26.1	20.0/24.6	18.4/22.3	21.4/26.0
Number of Atoms				
Protein	4,086	8,300	2,990	1,949
Water	42	170	177	0
Temperature factors				
Main Chain	54.7	49.8	34.4	100.8
Side Chain	64.2	58.4	37.1	103.4
LIR peptide	50.7	62.4	30.6	110.4
Solvent	37.6	40.2	37.5	-
Ramachandran Plot				
Allowed regions	99.4	99.1	100.0	99.6
Outliers	0.61	0.9	-	0.41
R.m.s.d from ideality				
Bond lengths (Å)	0.006	0.004	0.008	0.003
Planarity (°)	0.005	0.004	0.005	0.004
Angles (°)	1.077	0.732	1.040	0.778

* Co-crystals

Appendix Table S2. Angles between α2 and β2 in the mATG8s complexes with PLEKHM1 LIR.

mATG8	Angle (˚)
LC3A	-39.5
LC3B	-43.4
LC3C	-41.7
GABARAP	-34.2
GABARAP-L1	-35.6

Appendix Table S3. Distances (averaged across all monomers in an asymmetric unit (ASU)) between PLEKHM1-LIR residues (W635 and V638) and residues within mATG8-proteins. The table also shows the distances across the two families for an equivalent interaction (row means) and mean distance of each interaction within the respective pocket for each protein (column mean). Highlighted in red are the shortest distances, which are predominantly in the GABARAP-family members, demonstrating that the interactions in HP1 and HP2 pockets are generally shorter in the GABARAP-subfamily proteins.

Interaction of PLEKHM1 W635 with HP1 pocket (bond length measured in Å)

LC3A		LC3B		LC3C		LC3 family Mean	GABARAP		GABARAPL1		GABARAP family Mean
E19	3.52±0.02	*D19*	6.2±0.1	*E25*	4.50±0.03	***4.7***	*E17*	3.5±0.1	*E17*	4.3±0.9	***3.9***
I23	4.1±0.1	*I23*	4.4±0.3	*I29*	4.4±0.2	***4.3***	*I20*	4.7±0.9	*I20*	4.4±0.1	***4.5***
L53	5.0±0.12	*L53*	4.3±0.1	*L59*	5.00±0.02	***4.8***	*L50*	3.9±0.2	*L50*	4.3±0.5	***4.1***
F108	4.0±0.1	*F108*	4.2±0.1	*F114*	4.5±0.1	***4.2***	*F104*	3.50±0.03	*F104*	3.7±0.2	***3.6***
**Mean**	***4.2***		***4.8***		***4.6***			***3.9***		***4.2***	

Interaction of PLEKHM1 V638 with HP2 pocket (bond length measured in Å)

LC3A		LC3B		LC3C		LC3 family Mean	GABARAP		GABARAPL1		GABARAP family Mean
V54	4.20±0.03	*V54*	4.60±0.03	*V60*	4.6±0.1	***4.5***	*V51*	3.8±0.1	*V51*	4.3±0.3	***4.1***
V58	5.5±025	*V58*	5.4±0.2	*L64*	4.3±0.2	***5.1***	*L55*	4.6±0.1	*L55*	4.8±0.3	***4.7***
I66	3.4±0.1	*I66*	4.1±0.2	*I72*	4.5±0.1	***4***	*L63*	3.50±0.03	*L63*	3.9±0.2	***3.7***
**Mean**	***4.4***		***4.7***		***4.5***			***4.0***		***4.4***	

Appendix Table S4. Thermodynamic parameters of interactions between PLEKHM1-LIR peptide and mutated LC3B and GABARAP proteins (K30R and R28K, respectively).

	**ΔH** kcal mol^{-1}	**ΔS** cal mol^{-1} K^{-1}	**-T*ΔS** kcal mol^{-1}	**ΔG** kcal mol^{-1}	**K_A** $*10^6$ M^{-1}	**K_D** μM	**N**
LC3B wt	-5.8±0.2	+4.27	-1.27	-7.09	0.16±0.01	6.33	1.06±0.01
LC3B K30R	-6.1±0.2	+3.61	-1.08	-7.20	0.19±0.01	5.26	1.11±0.02
G_{ABARAP} wt	-10.60±0.1	-6.92	+2.06	-8.54	1.8±0.1	0.55	1.00±0.01
G_{ABARAP} R28K	-10.55±0.1	-6.71	+2.00	-8.55	1.9±0.1	0.54	1.05±0.01

Appendix Figure S1.

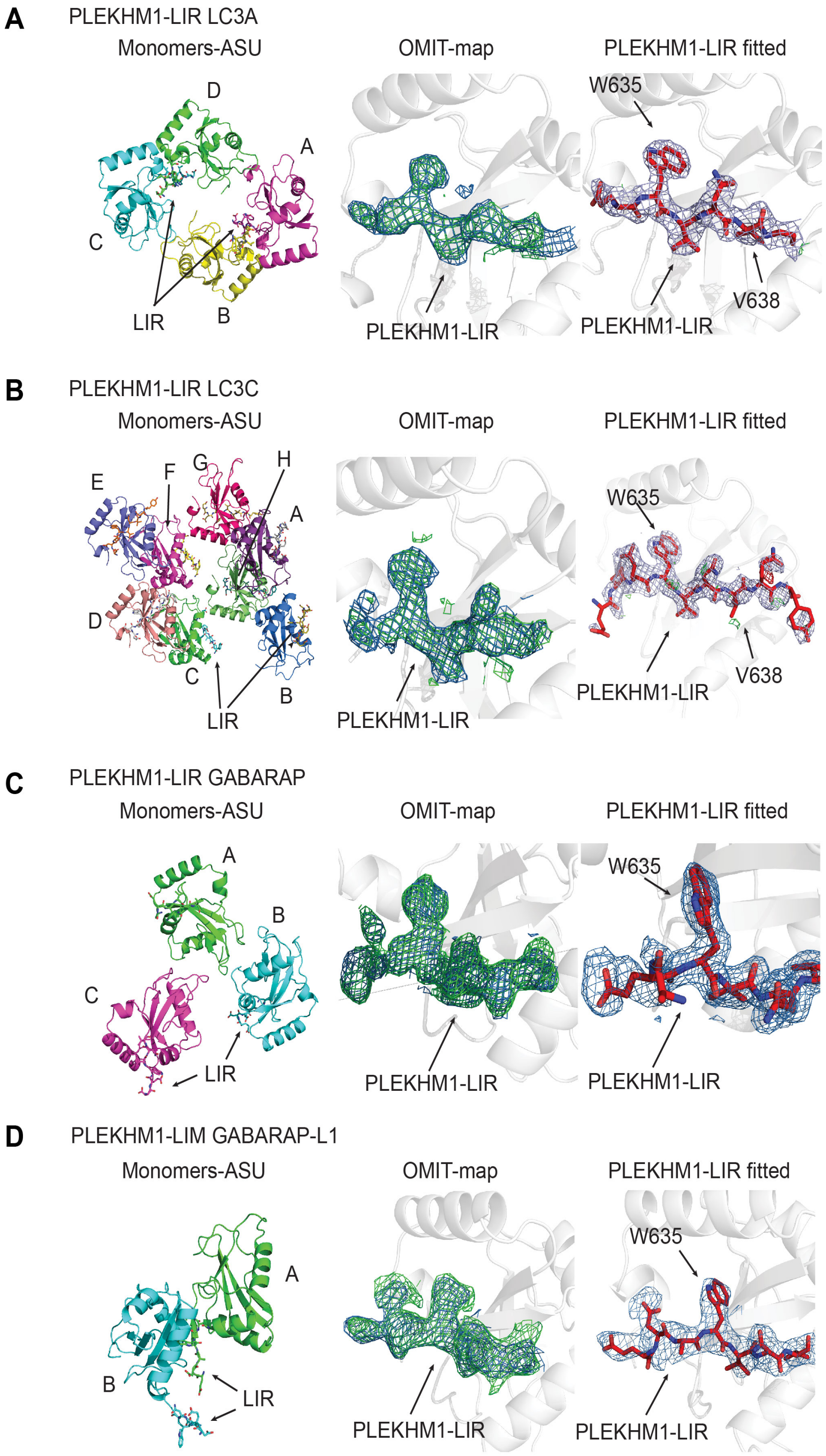

Appendix Figure S1. Structural analysis of the complexes between PLEKHM1-LIR peptide and mATG8 proteins.

A. Packing of four PLEKHM1$^{629-638}$-LC3A$^{2-121}$ monomers in asymmetric unit (left); omit map of the PLEKHM1-LIR electron density on the symmetry-related LC3A molecule (middle); omit map of the

PLEKHM1-LIR electron density on the symmetry-related LC3A molecule with the fitted PLEKHM1-LIR residues (right).

B. Packing of eight LC3C$^{8\text{-}125}$:PLEKHM1$^{629\text{-}642}$ monomers in asymmetric unit (left); omit map of the PLEKHM1-LIR electron density on the symmetry-related LC3C molecule (middle); omit map of the PLEKHM1-LIR electron density on the symmetry-related LC3C molecule with the fitted PLEKHM1-LIR residues (right).

C. Packing of three PLEKHM1$^{629\text{-}638}$-GABARAP$^{2\text{-}117}$ monomers in asymmetric unit (left); omit map of the PLEKHM1-LIR electron density on the symmetry-related GABARAP molecule (middle); omit map of the PLEKHM1-LIR electron density on the symmetry-related GABARAP molecule with the fitted PLEKHM1-LIR residues (right).

D. Packing of two PLEKHM1$^{629\text{-}638}$-GABARAP-L1$^{2\text{-}117}$ monomers in asymmetric unit (left); omit map of the PLEKHM1-LIR electron density on the symmetry-related GABARAP-L1 molecule (middle); omit map of the PLEKHM1-LIR electron density on the symmetry-related GABARAP-L1 molecule with the fitted PLEKHM1-LIR residues (right).

Appendix References

1. Wild P, Farhan H, McEwan DG, Wagner S, Rogov VV, Brady NR, Richter B, Korac J, Waidmann O, Choudhary C*, et al.* (2011) Phosphorylation of the autophagy receptor optineurin restricts Salmonella growth. *Science* **333**: 228-33
2. Novak I, Kirkin V, McEwan DG, Zhang J, Wild P, Rozenknop A, Rogov V, Lohr F, Popovic D, Occhipinti A*, et al.* (2010) Nix is a selective autophagy receptor for mitochondrial clearance. *EMBO Rep* **11**: 45-51
3. Rozenknop A, Rogov VV, Rogova NY, Lohr F, Guntert P, Dikic I, Dotsch V (2011) Characterization of the interaction of GABARAPL-1 with the LIR motif of NBR1. *J Mol Biol* **410**: 477-87
4. Rogov VV, Suzuki H, Fiskin E, Wild P, Kniss A, Rozenknop A, Kato R, Kawasaki M, McEwan DG, Lohr F*, et al.* (2013) Structural basis for phosphorylation-triggered autophagic clearance of Salmonella. *Biochem J* **454**: 459-66
5. Noda NN, Ohsumi Y, Inagaki F (2010) Atg8-family interacting motif crucial for selective autophagy. *FEBS Lett* **584**: 1379-85
6. Mohrluder J, Schwarten M, Willbold D (2009) Structure and potential function of gamma-aminobutyrate type A receptor-associated protein. *FEBS J* **276**: 4989-5005
7. Genau HM, Huber J, Baschieri F, Akutsu M, Dotsch V, Farhan H, Rogov V, Behrends C (2015) CUL3-KBTBD6/KBTBD7 ubiquitin ligase cooperates with GABARAP proteins to spatially restrict TIAM1-RAC1 signaling. *Mol Cell* **57**: 995-1010
8. Dougherty DA (1996) Cation-pi interactions in chemistry and biology: a new view of benzene, Phe, Tyr, and Trp. *Science* **271**: 163-8
9. Rogov V, Dotsch V, Johansen T, Kirkin V (2014) Interactions between autophagy receptors and ubiquitin-like proteins form the molecular basis for selective autophagy. *Mol Cell* **53**: 167-78
10. Drozdetskiy A, Cole C, Procter J, Barton GJ (2015) JPred4: a protein secondary structure prediction server. *Nucleic Acids Res* **43(W1)**: W389-94
11. Petherick KJ, Williams AC, Lane JD, Ordóñez-Morán P, Huelsken J, Collard TJ, Smartt HJ, Batson J, Malik K, Paraskeva C, *et al.* (2013) Autolysosomal β-catenin degradation regulates Wnt-autophagy-p62 crosstalk. *EMBO J* **32**: 1903-16
