# Supplementary material for: Structural and functional analysis of the GABARAP interaction motif (GIM)
Source: EMBO Rep. 2017 Jun 27;18(8):1382–96. doi: 10.15252/embr.201643587 (PMC5538626; doi:10.15252/embr.201643587)
Supplement: Supplementary file 2 — Expanded View Figures PDF [file EMBR-18-1382-s002.pdf]

Expanded View Figures

Figure EV1. Peptide array reveals preferences of particular mATG8 proteins to the 30 validated LIRs.

A Schematic presentation of the peptide array. Biotinylated LIR peptides were immobilized on the streptavidin-coated 96-well plate surface and treated with 6xHis-tagged ATG8 proteins. To visualize binding, HRP-coupled α-HIS antibody solution was passed over the complexes and level of binding was measured as a light absorption at 450 nm (solely mediated by HRP-coupled α-HIS antibodies).

B Relative preferences of LC3B and LC3C proteins to the 30 LIR motifs tested. Absorbance of LC3C divided by absorbance of LC3B (cyan bars) and absorbance of LC3B divided by absorbance of LC3C (magenta bars) to define whether each LIR shows preference towards either LC3C or LC3B protein. Values are mean of n = 3 independent experiments ± SEM.

C Relative preferences of GABARAP and GABARAP-L2 proteins to the 30 LIR motifs tested. Absorbance of GABARAP divided by absorbance of GABARAP-L2 (cyan bars) and absorbance of GABARAP-L2 divided by absorbance of GABARAP (green bars) to define whether each LIR shows preference towards either GABARAP or GABARAP-L2 proteins. Values are mean of n = 3 independent experiments ± SEM.

© 2017 The Authors

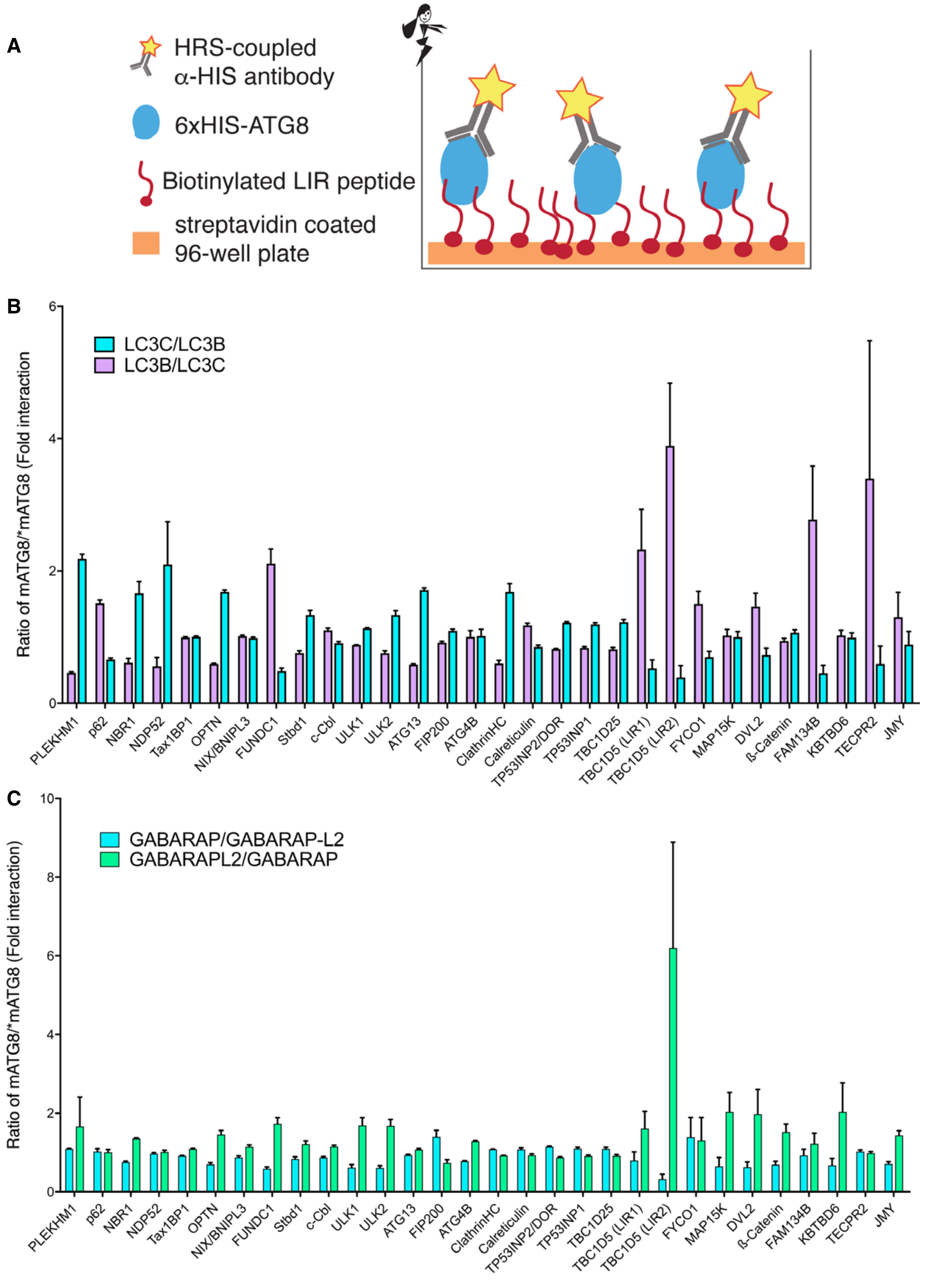

Figure EV1.

© 2017 The Authors

Figure EV2. Interactions between the GABARAP and LC3 family proteins with PLEKHM1-LIR peptide studied by NMR spectroscopy.

A Comparison of enthalpy (ΔH) and entropy (-T*ΔS) contribution to the total Gibbs energy (ΔG) for the binding of all hATG8 proteins to PLEKHM1-LIR and LC3B:p62-LIR as a control (last group of bars). Green colour of the bars indicates favourable contribution, and red indicates unfavourable contribution.

B Left panel: Representative sections of HSQC spectra for ^{15}N-labelled proteins (LC3B and GABARAP-L1) upon titration with PLEKHM1-LIR peptide are shown. Both plots show "fingerprint regions" of the proteins spectra (around HN resonance of K51 in LC3B, K48 in GABARAP-L1). Molar ratios of protein:peptide are rainbow colour-coded (1:0, 1:0.25, 1:0.5, 1:1, 1:2, 1:4; red to violet) for each titration step. Right panel: W635 side chain Hε1 resonance in PLEKHM1-LIR HSQC spectra is shown at different stages of titration with LC3 and GABARAP family proteins (here, LC3B and GABARAP). Molar ratio is given in the plots.

C CSP values (Δδ) at the last titration stages for LC3A, LC3B, GABARAP-L1 and GABARAP-L2 proteins are plotted against residue numbers. The solid lines indicate the standard deviations (σ) over all residues within each data set, and the dashed lines indicate double σ values.

D CSP values mapped on the protein structures (ribbon diagrams, PDB IDs: LC3A, 3ECI; LC3B, 1UGM; GABARAP-L1, 2R2Q; and GABARAP-L2, 1EO6). Residues with small ($\Delta\delta < \sigma$), intermediate ($\sigma < \Delta\delta < 2\sigma$) or strong ($2\sigma < \Delta\delta$) CSPs are marked in grey, yellow and red, respectively.

E CSP induced by titration of ^{15}N-labelled PLEKHM1-LIR peptide with LC3B (left) and GABARAP (right) proteins. Top panels: The overlaid ^{1}H-^{15}N HSQC spectra of free PLEKHM1 LIR peptide (red) and the peptide in presence of protein excess (green, four times molar ratio of LC3B and two times molar ratio for GABARAP). HN resonance assignments are given, and most prominent CSP are highlighted by dashed arrows. Bottom panels: The overlaid glutamine and asparagine side chain resonance area of PLEKHM1-LIR peptide HSQC spectra (the colour code is the same as above). The only δ2 H_2N resonances of N673 are significantly perturbed (green dashed arrow).

© 2017 The Authors

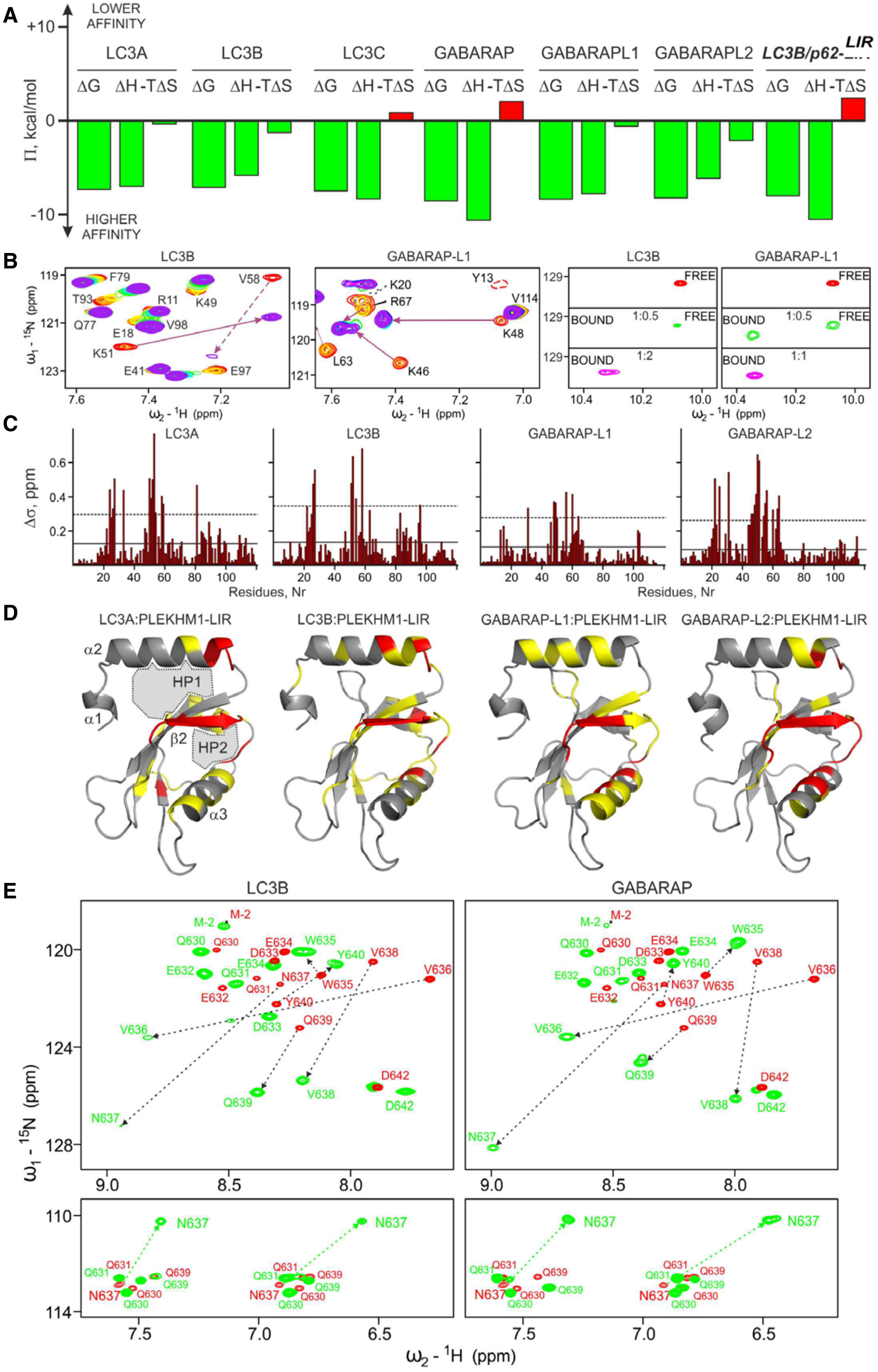

Figure EV2.

 © 2017 The Authors

Figure EV3. Comparative structural analysis of the PLEKHM1-LIR:mATG8 complexes.

A Overlay of monomers in an asymmetric unit of each complex structure. The four monomers in asymmetric unit of PLEKHM1$^{630–638}$-LC3A$^{2–121}$ overlay with RMSD of 0.174 Å. The PLEKHM1-LIR moiety represented in sticks is in the same orientation in all the monomers with the key amino acid W635 aligned well. Correspondingly, LC3B (data from McEwan *et al* [19]), LC3C, GABARAP and GABARAP-L1 monomers overlay with a similar accuracy (LC3B: 2 monomers, 0.217 Å; LC3C: 8 monomers, 0.426 Å; GABARAP: 3 monomers, 0.167 Å; GABARAP-L1: 2 monomers, 0.326 Å). Overlay of all five mATG8 monomer A structures in complex with PLEKHM1-LIR (purple) shows minor differences in the backbone conformation in the complexes. LC3A, PDB ID 5DPR, orange; LC3B, PDB ID 3X0W, red; LC3C, PDB ID 5DPW, yellow; GABARAP, PDB ID 5DPS, green; and GABARAP-L1, PDB ID 5DPR, cyan.

B Surface representation of LC3B in complex with PLEKHM1-LIR around the two hydrophobic pockets HP1 (left plot) and HP2 (right plot), marked yellow, deep core surface of each pockets are marked red. HP1 is formed between α_2 helix and β_2 strand and HP2 is formed between β_2, β_3 strands and α_3 helix. Specific LC3B residues contributing to the formation of HP1 and HP2 are indicated.

C Orientation of the α_2 helix in mATG8:PLEKHM1 complexes is specific for LC3 and GABARAP protein subfamilies. Left plot: Superimposition of all five mATG8 α_2 helices indicates different directions (directions are shown as arrows, and colour code is the same as in A). Remaining elements of the LC3A structure are shown in grey.

D Superimposition of LC3C and GABARAP-L1 α_2 helix (having biggest difference in orientation) in other projection (obtained from structures above after rotation in 90° around *x*-axis).

E Structural difference for the Loop 3 in all five solved mATG8–protein complexes with PLEKHM1-LIR. Overlay of all five mATG8–protein structures near Loop 3, and colour code is the same as in Fig 4A. Remaining elements of the LC3A structure are shown in grey.

F Structural difference for the Loop 6 in all five solved mATG8–protein complexes with PLEKHM1-LIR. Overlay of all five mATG8–protein structures near Loop 6, and colour code is the same as in Fig 4A. Remaining elements of the LC3A structure are shown in grey.

G Loop 6 gains in specificity in PLEKHM1-LIR interaction with LC3 and GABARAP subfamily members. Representative sections of HSQC spectra for ^{15}N-labelled proteins (LC3B, left plot; GABARAP-L1, right plots) upon titration with PLEKHM1-LIR peptide are shown. Red contours show resonances of the proteins in their free state, and purple contours show the resonances after saturation with PLEKHM1-LIR peptide (molar ratio 1:4 for LC3B and 1:2 for GABARAP-L1). Arrows show changes in position of the corresponding resonances. For LC3B, backbone HN resonances of residues within Loop 6 (S90 and S92, given in bold; V91 resonance remains invisible) show large CSP values and significant increase in intensity, indicating structural rearrangement and loss of flexibility for the Loop 6 upon PLEKHM1-LIR binding. In contrast, corresponding resonances of GABARAP-L1 residues (Q86, S87 in upper plot, and S88 in lower plot, in bold) show little CSP and no changes in intensity, indicating that Loop 6 in GABARAP family proteins is not significantly affected by binding of PLEKHM1-LIR. Thus, despite Loop 6 being located distantly from HP1 and HP2, it contributes significantly and specifically to the stabilization of mATG8:LIR complexes. According to our NMR experiments, in LC3 proteins, Loop 6 switches between unstructured (LIR-unbound; low signal intensities) and structured (LIR-bound; increased backbone HN resonance intensity), resulting in significant CSP for the HN resonances within the loop. However, for GABARAP proteins, the corresponding CSP are small, indicating this loop remains mostly structured in both unbound and LIR-bound state, highlighting differences between these related, but different families of mATG8–proteins.

© 2017 The Authors

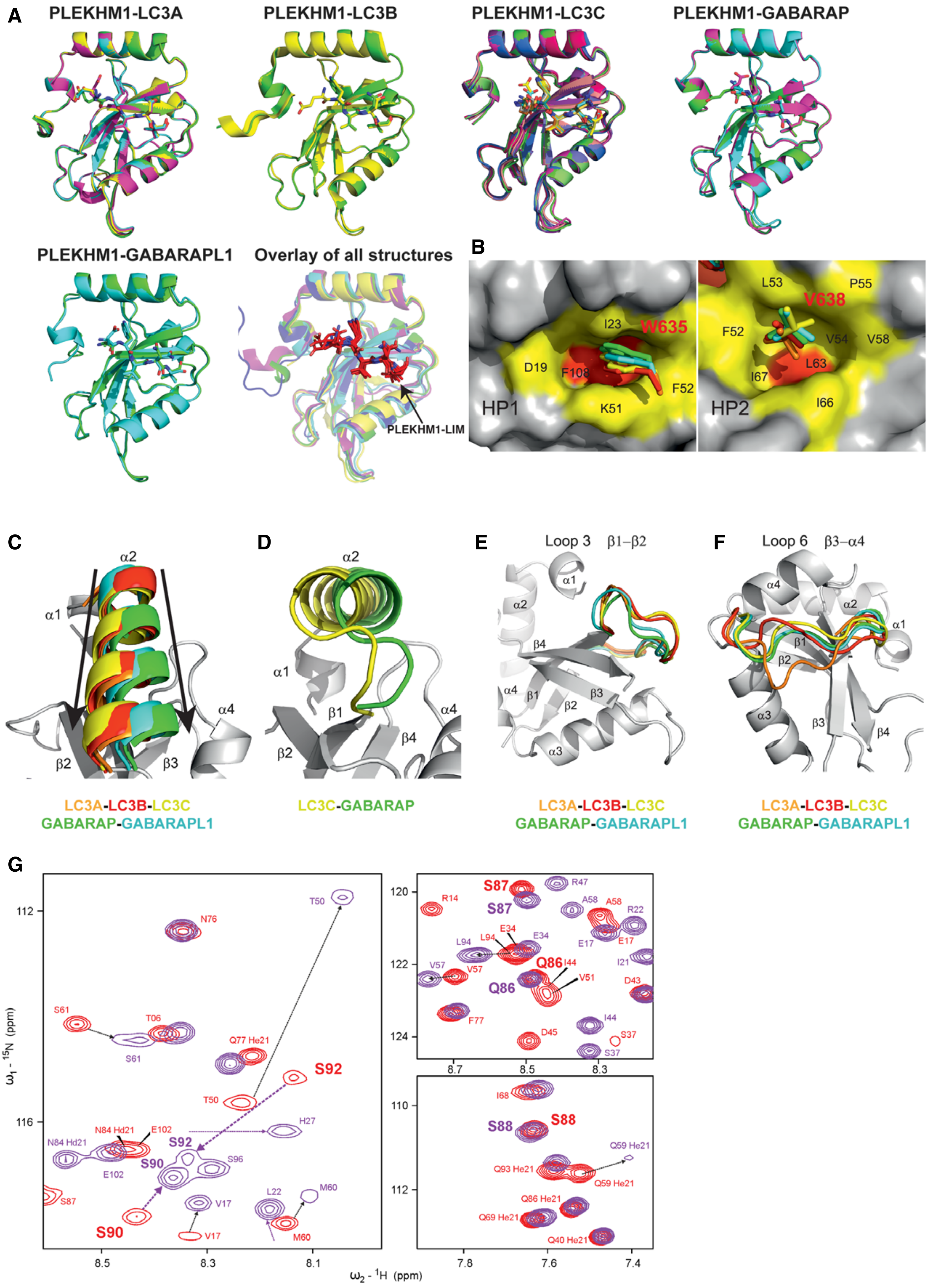

Figure EV3.

 © 2017 The Authors

Figure EV4. Detailed analysis of the microenvironments for PLEKHM1-LIR positions X_1 (V636) and X_2 (N637).

A Sections of complex structures representing V636 of PLEKHM1 and its microenvironments. In the LC3 subfamily proteins (left plot), R70/70/76 (LC3A/B/C) side chains are closer to the CG1/2 atoms of V638 (~3.6/3.7/4.1 Å), while for GABARAP subfamily (right plot) these distances are 4.6 Å (GABARAP) and 4.2 Å (GABARAP-L1). Due to this closer proximity, R70/70/76 polar groups become more fixed in their position and organize neighbouring negatively charged carboxyl groups of the D48/48/54 via salt bridges. In the GABARAPs complex structures, the corresponding R67 side chains are flexible and the salt bridges are absent. This is likely due the increased flexibility of the GABARAPs D45 residues, which are not visible in the electron density maps. Side chains of corresponding residues are shown as sticks with orange/red/yellow colour codes for LC3A/LC3B/LC3C and green/cyan for GABARAP/GABARAP-L1, respectively. V636 residue is shown as grey sticks. Salt bridges are shown as black dashed lines; GABARAPs D45 side chains, which are not present at the electron density maps, are shown as dashed cones. Structure of LC3B:PLEKHM1-LIR complex with indicated elements of the secondary structures is shown as a background for LC3 subfamily proteins, and structure of GABARAP:PLEKHM1-LIR complex is shown for GABARAP subfamily proteins.

B Sections of complex structures representing N637 of PLEKHM1 and its microenvironments. Left plot: Complete microenvironments of PLEKHM1 N637 (X_2 position) for each PLEKHM1-LIR complex. PLEKHM1 N637 and corresponding mATG8 residues (K30/30/36 and H27/H27/F33 for LC3A/B/C; R28/28 and Y25/25 for GABARAP/GABARAP-L1) are shown as sticks. Colour code is defined above. For the GABARAP subfamily protein complexes, the intermolecular hydrogen bond has better geometry (distances and angles). Additionally, in the GABARAP subfamily structures, this hydrogen bond is stabilized by coordinated cation–π interaction of R28 guanidinium moiety and corresponding Y25 aromatic side chain. Right plot presents this situation for GABARAP:PLEKHM1-LIR complex. Intermolecular hydrogen bond is shown as a black dotted line and cation–π interactions are shown as blue dashed lines. For the GABARAP subfamily structures, geometry of the corresponding K30/30/36 and H27/H27/F33 (for LC3A/B/C, respectively) is not suitable to form this type of stabilization.

C ITC titrations for LC3B K30R and GABARAP R28K mutants with PLEKHM1-LIR peptide (in comparison with that for WT proteins) are shown in the same scales as in Fig 2A. In each plot, the top diagrams display the raw measurements and the bottom diagrams show the integrated heat per titration step. Thermodynamic parameters are given in Appendix Table S4.

D Upper panels: The overlaid ^1H-^{15}N HSQC spectra of PLEKHM1-LIR peptide in the presence of LC3B WT (green) and LC3B K30R (blue) are shown at the left plot. Four times molar excess of the LC3B proteins over peptide were used in this experiment. Right: The overlaid ^1H-^{15}N HSQC spectra of PLEKHM1-LIR peptide in the presence of GABARAP WT (green) and GABARAP R28K (blue), two times molar excess of the proteins over peptide. HN resonances assignments are given; most prominent CSPs are highlighted by dashed arrows. Bottom panels: Corresponding representative sections of the ^1H-^{15}N HSQC spectra show asparagine and glutamine H_2N side chain resonances. The red contours show N637 $\delta 2$ H_2N resonances at initial state before titration; green dashed arrows represent CSP for the wild-type LC3B and GABARAP proteins, blue dashed arrows represent CSP for corresponding mutants (K30R for LC3B, and R28K for GABARAP).

© 2017 The Authors

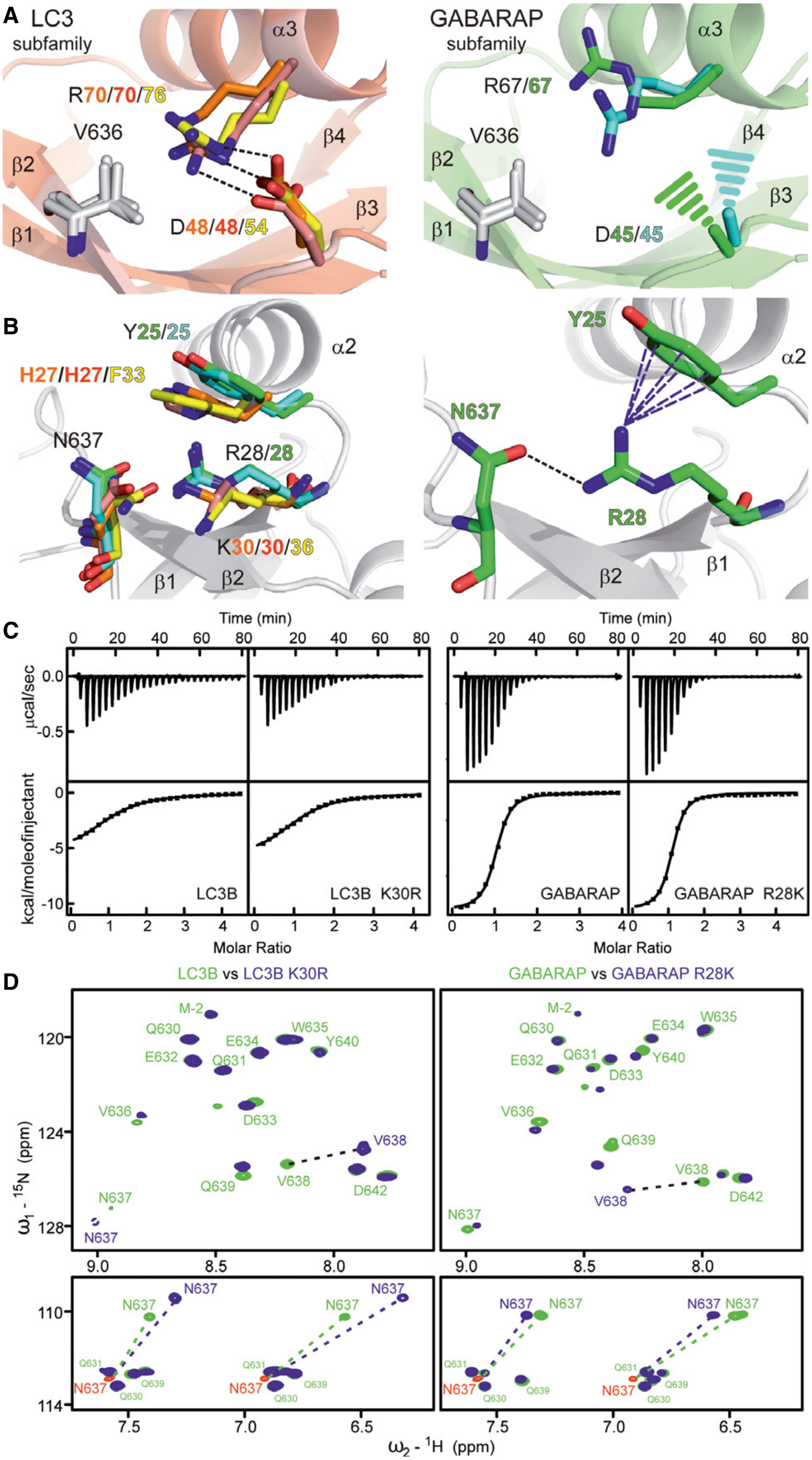

Figure EV4.

© 2017 The Authors

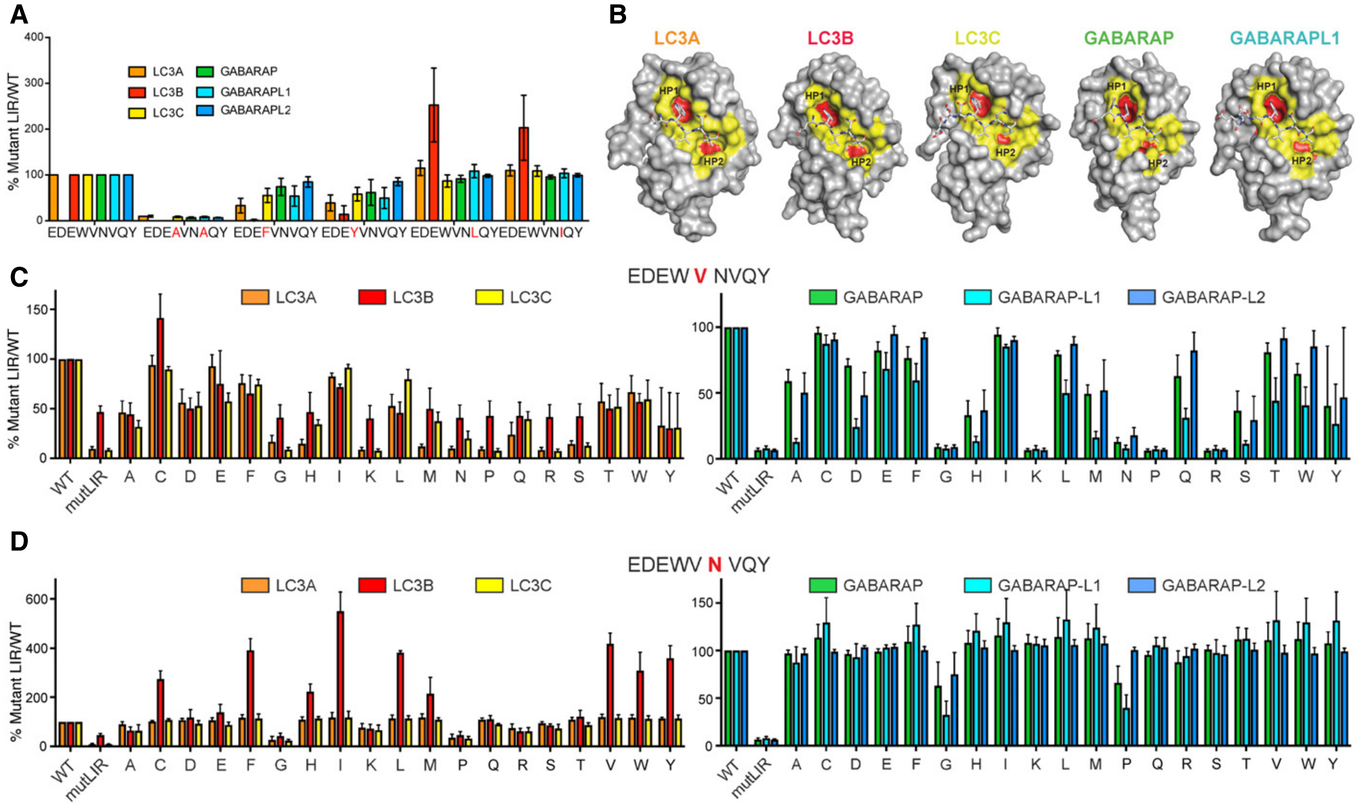

Figure EV5. Role of PLEKHM1-LIR residues in Θ and Γ positions and their accommodation in the HP1 and HP2 on surfaces of mATG8 proteins.

A Role of PLEKHM1-LIR residues in Θ and Γ positions revealed by mutational analysis. Biotinylated peptides of PLEKHM1-LIR WT (EDEWVNVQY), PLEKHM1-mutLIR (EDE**A**VN**A**QY) or W635F, W635Y, V638L or V638I were tested in their ability to interact with 6xHis-tagged LC3A, LC3B, LC3C GABARAP, GABARAP-L1 and GABARAP-L2 proteins. Absorbance values for each LC3/GABARAP protein were expressed as a fold change over WT LIR sequence to assess the impact of each mutation on the interaction. Bars represent mean ± SEM of *n* = 3 independent experiments.

B Surface representation of LC3A, LC3B, LC3C and GABARAP, GABARAP-L1 proteins in complex with PLEKHM1-LIR. Important residues around the two hydrophobic pockets HP1 and HP2 (marked red) are marked in yellow with the deep hydrophobic core surface of each pockets marked in red.

C Biotinylated peptides of PLEKHM1-LIR WT (EDEWVNVQY), PLEKHM1-mutLIR (EDE**A**VN**A**QY) or V636X, where X is all other amino acids (given by single-character code), were tested in their ability to interact with 6xHis-tagged LC3A, LC3B and LC3C (left plot) and 6xHis-tagged GABARAP, GABARAP-L1 and GABARAP-L2 proteins (right plot). Absorbance values for each LC3/GABARAP protein were expressed as a percentage over WT LIR sequence (100%) to assess the impact of each mutation on the interaction. Bars represent mean ± SEM of *n* = 3 independent experiments.

D Biotinylated peptides of PLEKHM1-LIR WT (EDEWVNVQY), PLEKHM1-mutLIR (EDE**A**VN**A**QY) or N637X, where X is all other amino acids (given by single-character code), were tested in their ability to interact with 6xHis-tagged LC3A, LC3B and LC3C (left plot) and 6xHis-tagged GABARAP, GABARAP-L1 and GABARAP-L2 proteins (right plot). Absorbance values for each LC3/GABARAP protein were expressed as a percentage over WT LIR sequence (100%) to assess the impact of each mutation on the interaction. Bars represent mean ± SEM of *n* = 3 independent experiments.
