# Supplementary material for: Structural and functional analysis of the GABARAP interaction motif (GIM)
Source: EMBO Rep. 2017 Jun 27;18(8):1382–96. doi: 10.15252/embr.201643587 (PMC5538626; doi:10.15252/embr.201643587)
Supplement: Supplementary file 4 — Source Data for Figure 2 [file EMBR-18-1382-s003.pdf]

Source Data for Figure 2

Fig 2B - X-Ray Film

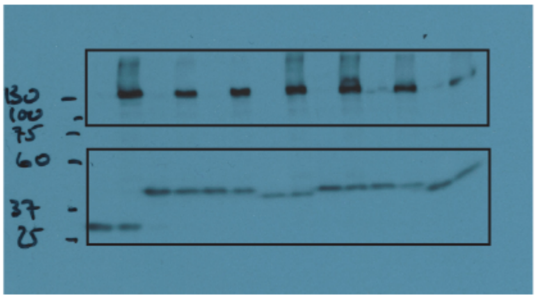

Anti-Flag (Input; Upper);
Anti-GFP (Input;lower)

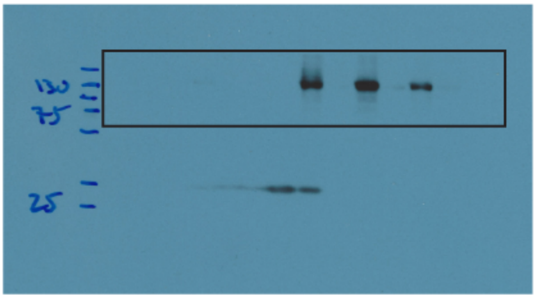

Anti-Flag (IP)

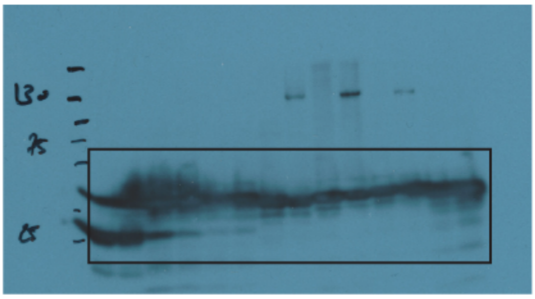

Anti-GFP (IP)

Fig 2C - BIORAD ChemiDoc

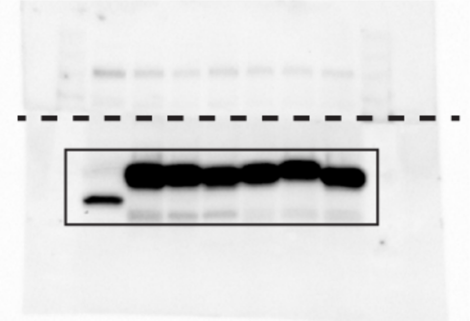

Anti-GFP (Input; Lower)

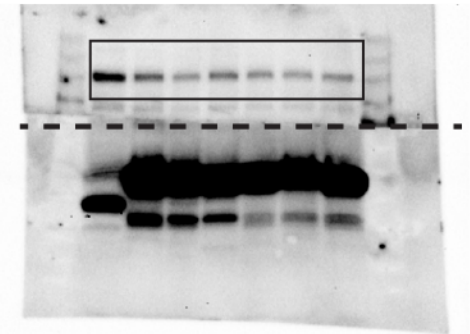

Anti-Plekhm1 (Input; Upper)

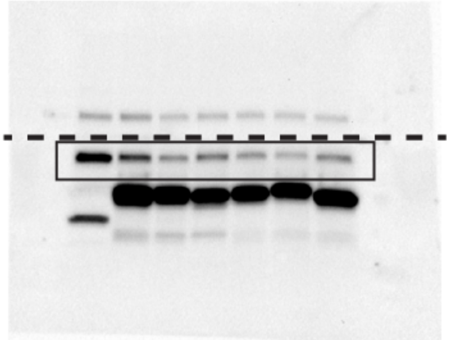

Anti-P62 (Input; lower)

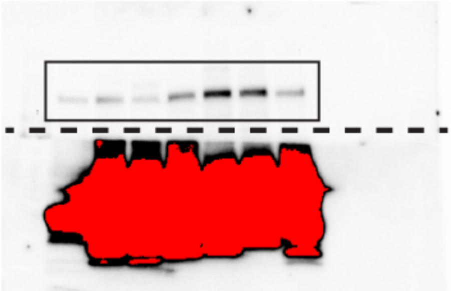

Anti-Plekhm1 (IP)

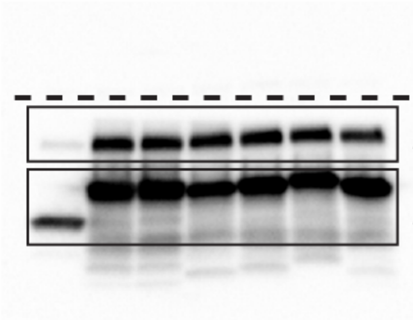

Anti-P62 (IIP)

Anti-GFP (IP)

Fig 2D - BIORAD ChemiDoc

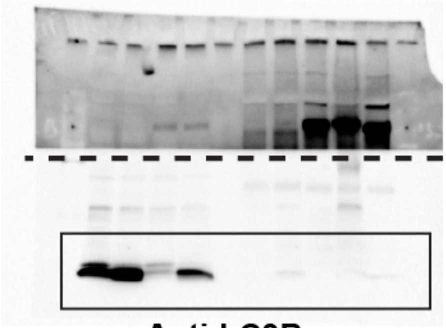

Anti-LC3B

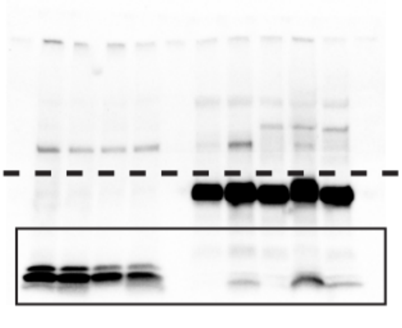

Anti-GABARAP

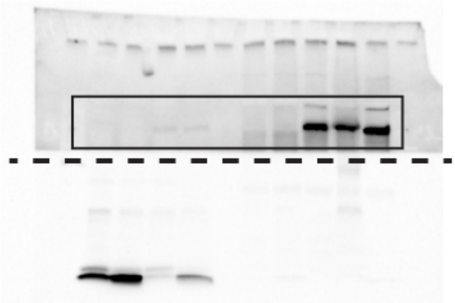

Anti-Plekhm1
