# Supplementary figures and images for: Structural and functional analysis of the GABARAP interaction motif (GIM)
Source: EMBO Rep. 2017 Jun 27;18(8):1382–96. doi: 10.15252/embr.201643587 (PMC5538626; doi:10.15252/embr.201643587)

Fig 4C - X-Ray Film

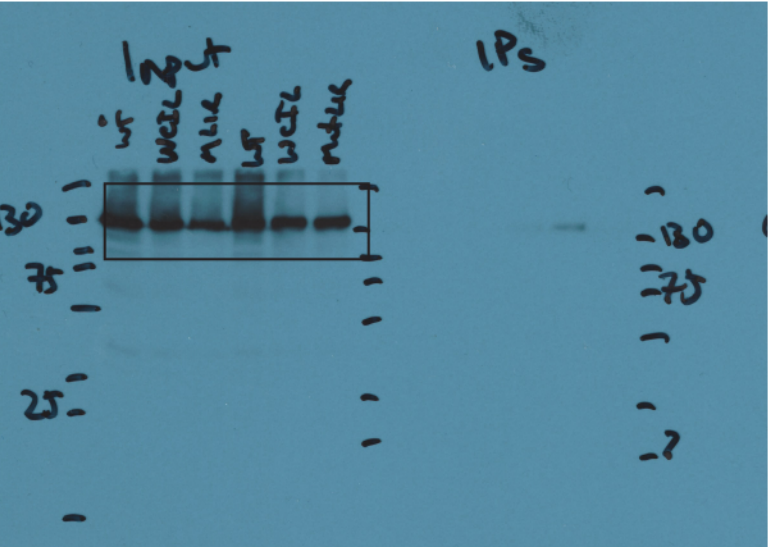

Anti-Flag (Input)

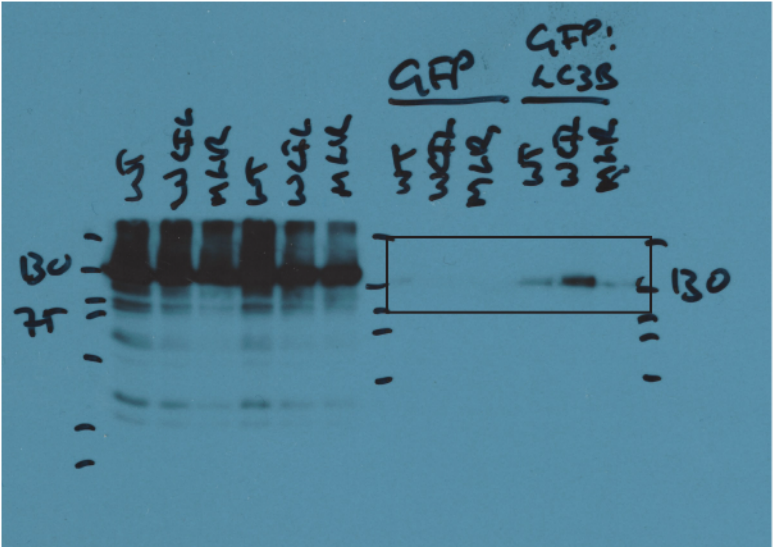

Anti-Flag (IP)

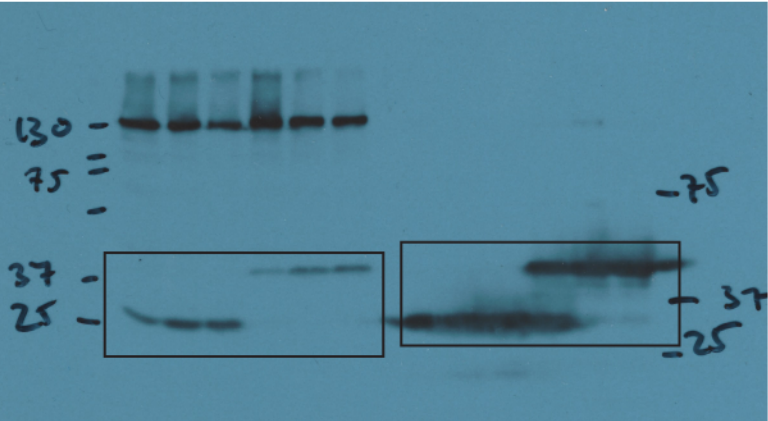

Anti-GFP(Input)

Anti-GFP(IP)

Supplement: Supplementary file 5 — Source Data for Figure 4 [file EMBR-18-1382-s004.pdf]
