# Supplementary material for: Structural and functional analysis of the GABARAP interaction motif (GIM)
Source: EMBO Rep. 2017 Jun 27;18(8):1382–96. doi: 10.15252/embr.201643587 (PMC5538626; doi:10.15252/embr.201643587)
Supplement: Supplementary file 6 — Source Data for Figure 5 [file EMBR-18-1382-s005.pdf]

Source Data for Figure 5

Fig 5B - X-Ray Film

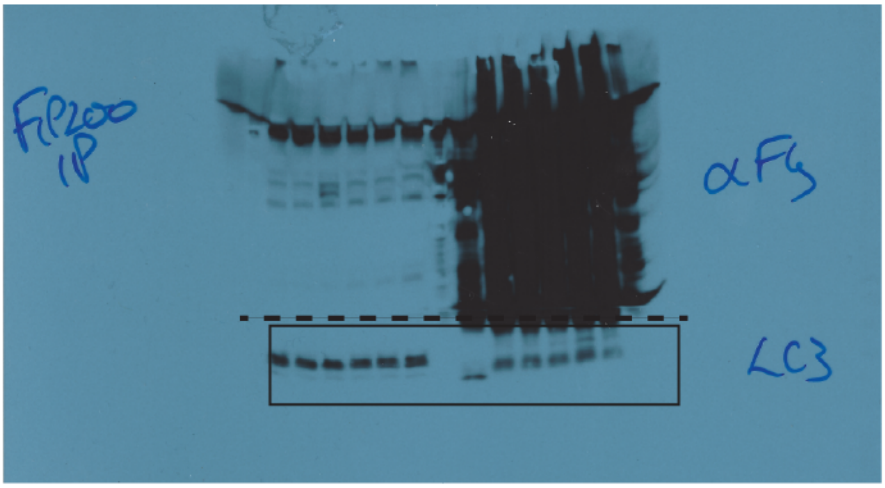

ANTI-LC3 (INPUT & IP)

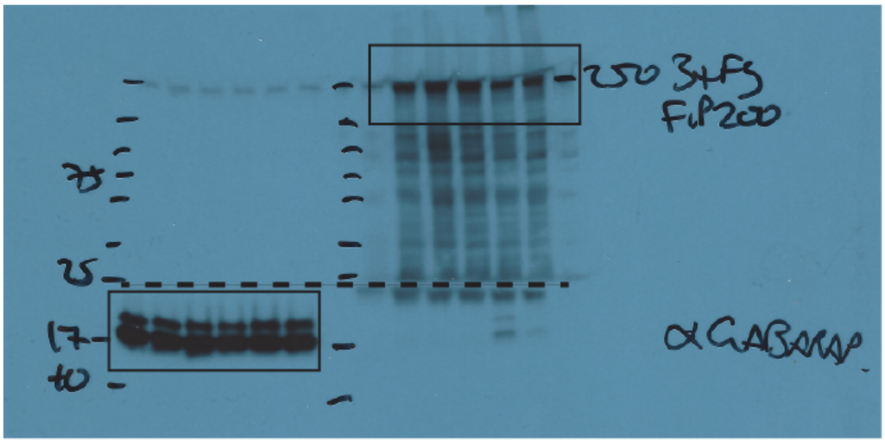

ANTI-FLAG (FIP200; IP)

Anti-GABARAP (Input)

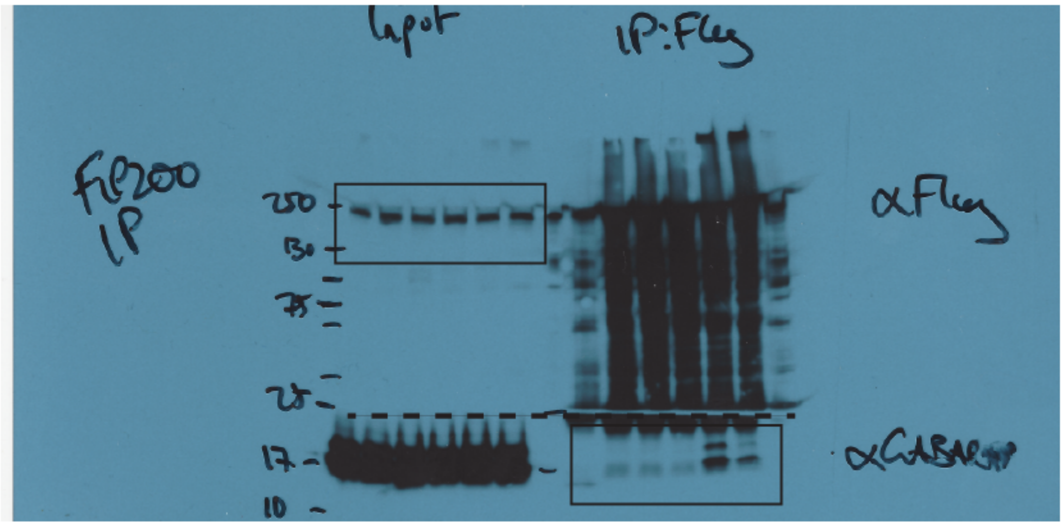

ANTI-FLAG (INPUT; FIP200)

Anti-GABARAP (IP)

Fig 5C- X-Ray Film

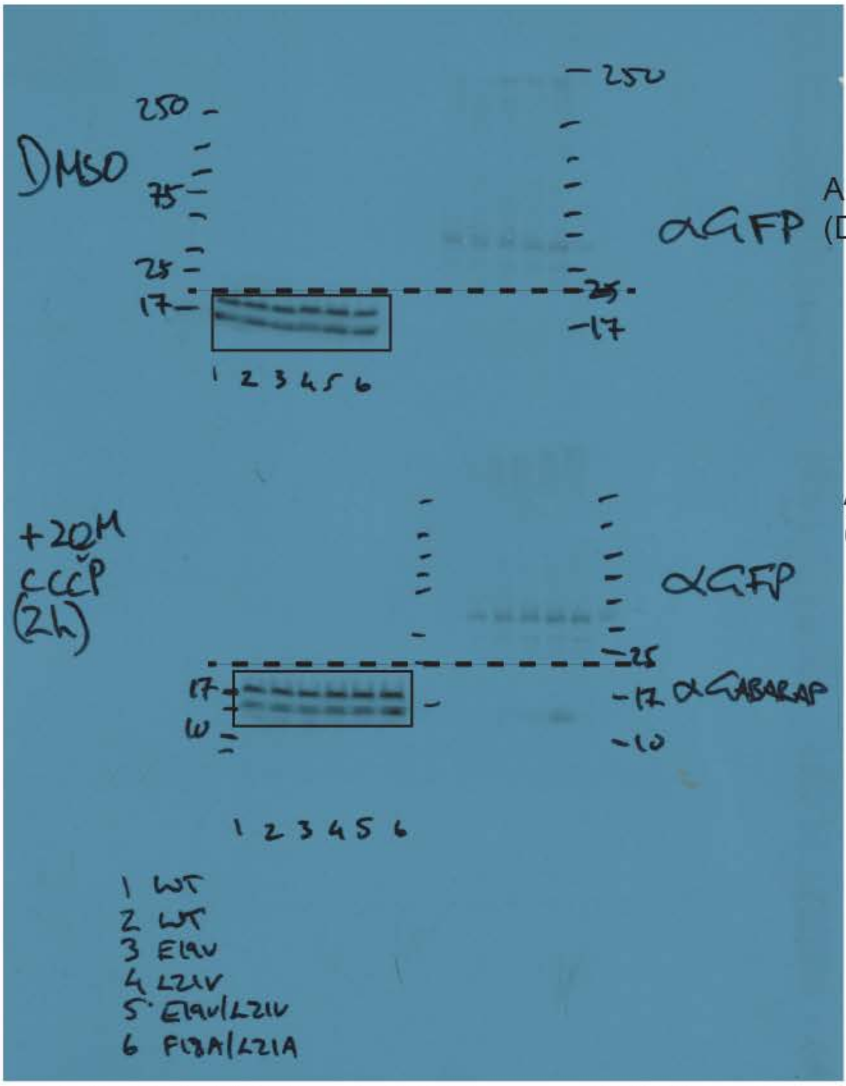

Anti-GABARAP (Input ;DMSO upper; CCCP lower)

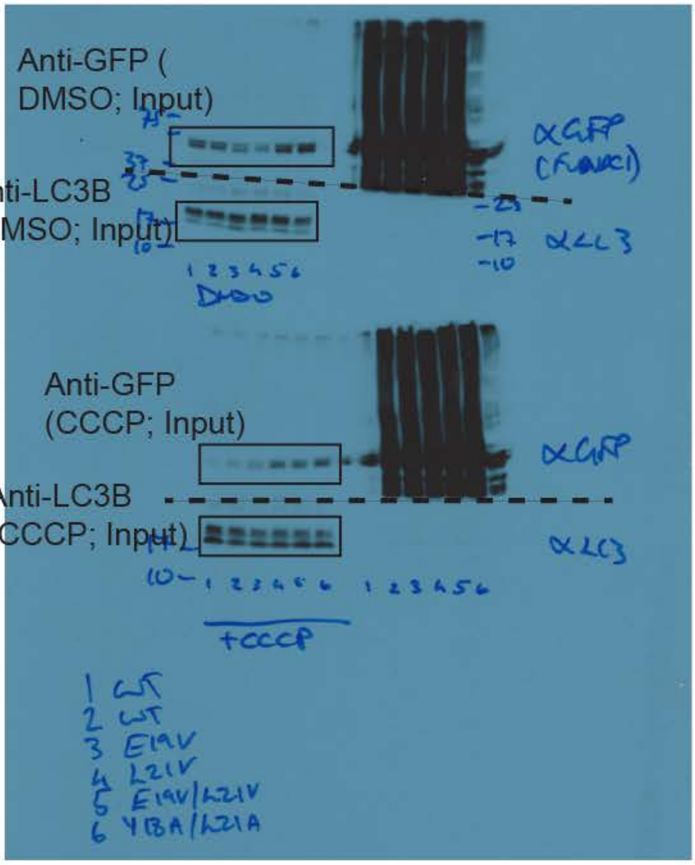

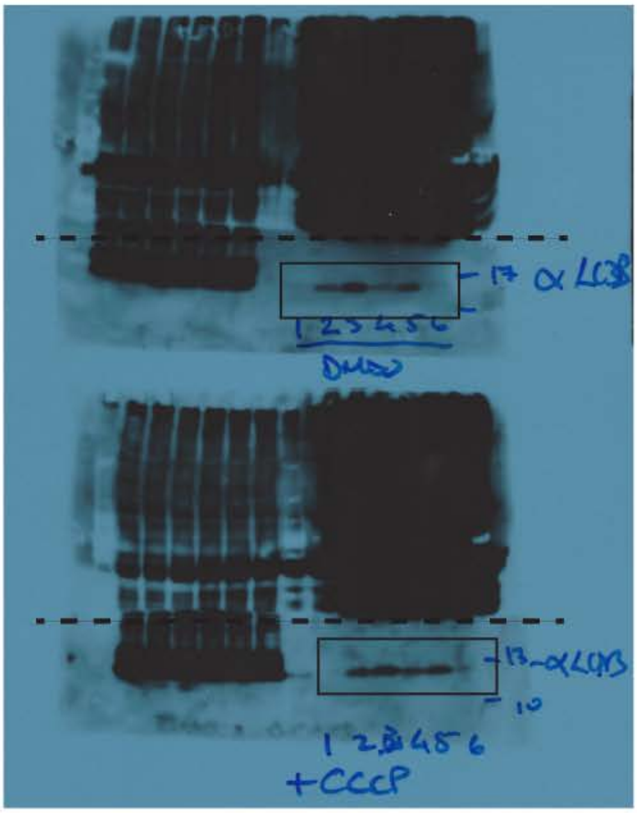

Anti-LC3B (IP;DMSO upper; CCCP lower)

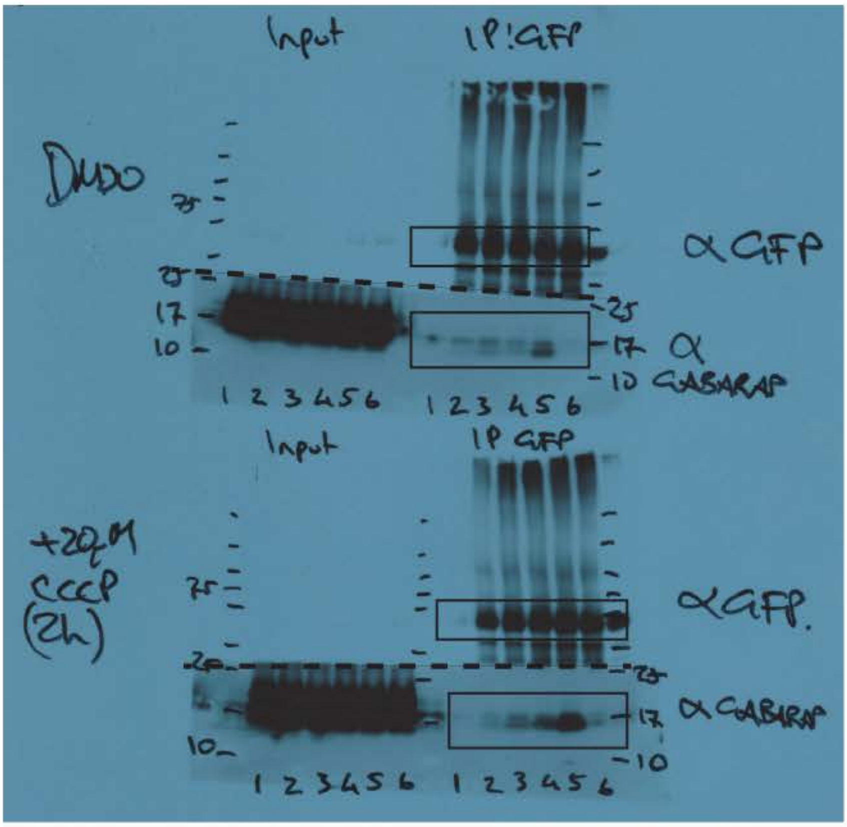

Anti-GFP (Input ;DMSO upper blot ; CCCP lower blot)
Anti-GABARAP (Input ;DMSO upperblot ; CCCP lower blot)
